# Patterns of progression differ between Kellgren-Lawrence 2 and 3 knees fulfilling different definitions of a cartilage-meniscus phenotype in the Foundation for National Institutes of Health Osteoarthritis Biomarkers study (FNIH)

**DOI:** 10.1016/j.ocarto.2022.100284

**Published:** 2022-06-09

**Authors:** Frank W. Roemer, Jamie E. Collins, David J. Hunter, Shadpour Demehri, Ali Guermazi

**Affiliations:** a Quantitative Imaging Center, Department of Radiology, Boston University School of Medicine, 820 Harrison Avenue, FGH Building, 4th Floor, Boston, MA, 02118, USA; b Department of Radiology, Universitätsklinikum Erlangen and Friedrich-Alexander University Erlangen-Nürnberg (FAU), Maximiliansplatz 3, 91054 Erlangen, Germany; c Orthopaedics and Arthritis Center of Outcomes Research, Brigham and Women's Hospital, Harvard Medical, School, 75 Francis Street, BTM Suite 5016 Boston, MA 02115, Boston, MA, USA; d Department of Rheumatology, Royal North Shore Hospital and Institute of Bone and Joint Research, Kolling Institute, University of Sydney, Sydney, Australia; e Russell H. Morgan Department of Radiology and Radiological Sciences, Johns Hopkins University, 601 N Caroline St, JHOC 4240, Baltimore, MD, 21287, USA; f Department of Radiology, VA Boston Healthcare System, 1400 VFW Parkway, Suite 1B105, West Roxbury, MA, 02132, USA

**Keywords:** Cartilage, Progression, Phenotype, MRI, Knee, Osteoarthritis

## Abstract

**Objective:**

Aim was to describe three definitions for an MRI-defined cartilage-meniscus phenotype and to report phenotypic progression in Kellgren-Lawrence (KL) 2 and 3 knees over 48 months.

**Methods:**

The study sample was a nested case-control study with knees showing either 1) radiographic and pain progression (“composite” case), 2) radiographic progression only, 3) pain progression only, and 4) no progression. MRI was performed on 3T systems. MRIs were read according to the MOAKS system. Knees were classified as having the cartilage-meniscus phenotype according to three modified ROAMES (Rapid OsteoArthritis MRI Eligibility Score) definitions (D): 1) ​≤ ​2.2 (10–75% of the region of cartilage surface area with 10–75% affected by full thickness loss), i.e. ‘D1’ 2) ≤ 2.1 (10–75% of the region of cartilage surface area with <10% affected by full thickness loss), i.e. ‘D2’ and 3) ≤ 2.0 (10–75% of the region of cartilage surface area without full thickness loss), i.e. ‘D3’. The odds of being a composite case for those with vs. without each definition was determined using logistic regression.

**Results:**

485 knees were included. For KL2 knees 191 (64%) knees fulfilled D1 criteria, 183 (62%) D2 and 167 (56%) D3. For KL3 these numbers were 164 (87%), 103 (55%) and 77 (41%). Odds for being a composite case for KL2 knees were 2.52 (95% CI 1.40,4.54) for D1, 1.93 (95% CI 1.11,3.35) for D2 and 1.92 (95% CI 1.13,3.28) for D3. For KL3 knees odds were 0.32 (95% CI 0.13,0.78) for D1, 0.56 (95% CI 0.31,1.01) for D2 and 0.49 (95% CI 0.26,0.91) for D3.

**Conclusion:**

Increased odds for progression are seen for KL2 knees for all definitions, while this was not observed for KL3 knees. KL3 knees exceeding the maximum damage thresholds and not fulfilling the phenotypic definitions are still likely to experience further progression.

## 1 Introduction

Imaging plays an important role in determining structural disease severity and potential eligibility of patients recruited to disease-modifying osteoarthritis drug (DMOAD) trials [1]. From a structural perspective, knees with established structural OA grades 2 and 3 on the Kellgren-Lawrence (K-L) scale are commonly considered eligible. In the context of personalized medicine, it has been recognized that OA is a heterogeneous disease. This heterogeneity led to the concept of defining different subtypes of OA, also known as phenotypes, that are characterized by distinct underlying pathobiological and pain mechanisms [2]. While imaging-based structural characterization into subtypes of disease is still in its infancy, three main MRI-defined structural phenotypes have been proposed, i.e., inflammation, cartilage-meniscus and subchondral bone [3]. Acknowledging that these subtypes may not necessarily fulfill the overarching suggested definition of OA phenotypes including clinical parameters [2], structural stratification may be relevant particularly in the context of clinical trials. Recently introduced Rapid OsteoArthritis MRI Eligibility Score (ROAMES) system allows structural classification based on abbreviated MRI assessment and thus, may potentially be applicable in screening efforts for inclusion into DMOAD trials [4]. In a recent report based on the Foundation for National Institutes of Health (FNIH) Osteoarthritis Biomarkers Consortium cohort, we found that the bone phenotype was associated with increased odds of progression, while the inflammatory and cartilage-meniscus phenotypes were not [5]. However, a drawback of that analysis was the low prevalence of the phenotype yield using the primary definitions according to ROAMES. This was the case particularly for the inflammatory and cartilage/meniscus phenotypes (prevalence <5% for both phenotypes) [4]. Concerning progression, the cartilage-meniscus phenotype is of particular relevance given the target tissue of most DMOAD approaches is cartilage - either by anti-catabolic or anabolic pathways.

Thus, the aim of our study was to describe frequencies for different structural thresholds regarding the definition of a modified ROAMES cartilage-meniscus phenotype and to report patterns of progression in Kellgren-Lawrence (KL) 2 and 3 knees over 48 months in a simulated clinical trial population, the FNIH cohort.

## 2 Methods

### 2.1 Sample selection

The FNIH study is a nested case-control study embedded within the larger Osteoarthritis Initiative (OAI) study [6]. In brief, the OAI is a multicenter prospective observational cohort study of knee OA (https://oai.nih.gov) that enrolled 4796 participants aged 45–79 years at four clinical centers. Details of the OAI inclusionary and exclusionary criteria have been published [7].

### 2.2 Case-control selection

The FNIH study sample was defined by symptomatic and structural progression outcomes over 48 months [1]. A pre-determined number of index knees was selected in the following outcome groups, for measurement of imaging biomarkers: 1) case knees had both radiographic and pain progression (i.e. ‘composite case’); 2) knees with radiographic but not pain progression (i.e., joint space loss - ‘JSL case’), 3) knees with pain but not radiographic progression, and 4) knees with neither radiographic nor pain progression.

Knees with KL1 at baseline were excluded in the current analysis as these are not considered to have radiographic OA. Knees with posterior meniscal root tears at baseline were also excluded as these knees are considered at increased risk for rapid structural progression due to the effect of a subtotal meniscectomy biomechanically and increased risk for subchondral insufficiency fractures [[8], [9], [10], [11]].

### 2.3 MRI acquisition

MRIs of both knees were acquired using 3T systems (Siemens MAGNETOM Trio, Erlangen, Germany) at the four OAI clinical sites. The sequence protocol included a coronal intermediate-weighted 2-dimensional turbo spin echo sequence, a sagittal 3-dimensional dual-echo steady-state (DESS) sequence, and a sagittal intermediate-weighted fat-suppressed turbo spin-echo sequence [12]. Two musculoskeletal radiologists, blinded to clinical data and case-control status, read the baseline MRIs according to the MRI Osteoarthritis Knee Score (MOAKS) system [13].

### 2.4 Phenotypic definitions

The current analysis focuses primarily on the medial compartment, which is commonly the primary outcome in clinical DMOAD trials [[14], [15], [16]]. Three different phenotypes were defined according to the maximum severity of cartilage damage in the medial compartment. To be classified in a phenotype, knees had to have damage in at least one subregion (score >0) but maximum damage not exceeding the following thresholds in any of the five medial subregions: 1) ​≤ ​2.2 (10–75% of the region of cartilage surface area with 10–75% affected by full thickness loss), i.e. ‘D1’ 2) ≤ 2.1 (10–75% of the region of cartilage surface area with <10% affected by full thickness loss), i.e. ‘D2’ and 3) ≤ 2.0 (10–75% of the region of cartilage surface area without full thickness loss), i.e. ‘D3’. These definitions will result in the exclusion of compartments without any damage and those with severe wide-spread full thickness damage of more than 75%. In a separate analysis, we defined a combined medial and lateral cartilage phenotype based on the concomitant presence of the same criteria for both the medial and lateral compartments as described above.

### 2.5 Statistical analysis

For each of the three definitions, the odds of outcome (composite or JSL case) for those with vs. without the phenotype according to threshold was determined using logistic regression. Knees not fulfilling the definition were used as the referent. Sensitivity analyses focused on the reference knees not fulfilling these phenotypic definitions to understand proportions of knees with either “too much” (i.e., max severity greater than threshold) or “no” damage (i.e., no subregions with score >0). In addition, we present the frequencies of knees using different cartilage-meniscus phenotype definitions based on medial-only or medial/lateral compartment cartilage thresholds. Further, we are presenting data on the frequencies of knees using the different cartilage-meniscus phenotype definitions based on medial cartilage thresholds and concomitant ipsicompartmental presence of any or severe medial meniscal damage. All analyses were conducted in SAS 9.4 (SAS Institute, Cary NC).

## 3 Results

After exclusion of KL 1 knees (n ​= ​71) and knees with posterior meniscal root tears (n ​= ​44), 485 knees were included. Excluded participants were similar to included participants with respect to baseline characteristics and case status. Mean age of the participants was 61 years (standard deviation (SD) ​± ​8.8), 59% of the participants were women, average body mass index was 31.0 ​kg/m^2^ (SD ​± ​4.8). 297 knees were KL2 and 188 knees KL3.

For KL2 knees, 191 (64%) knees fulfilled D1 criteria. Of the 106 KL2 knees that did not meet the criteria, 5 (2%) exceeded the maximum damage severity threshold and 101 (34%) had no damage in any medial subregion. 183 (62%) met D2 criteria and 167 (56%) D3. For KL3 these numbers were 164 (87%) for D1, 103 (55%) for D2 and 77 (41%) for D3. Of the 24 KL3 knees that did not meet the D1 criteria, 23 (12%) exceeded the maximum damage severity threshold and 1 (1%) knee had no damage in any medial subregion. Odds for being a composite case were 2.52 (95% CI 1.40,4.54) for D1, 1.93 (95% CI 1.11,3.35) for D2 and 1.92 (1.13,3.28) for D3. For KL3 knees odds were 0.32 (95% CI 0.13,0.78) for D1, 0.56 (95% CI 0.31,1.01) for D2 and 0.49 (95% CI 0.26,0.91) for D3. For the JSL-only outcome numbers were similar, with increased odds for KL2 knees and a protective effect for KL3 knees (Table 1). For KL2 knees, 2% (D1), 4% (D2) and 10% (D3) of the controls had too much damage, while 34% did not have any damage. For KL3 12%, 45% and 59% had too much damage, while only one (1%) knee had no damage in the medial compartment. Further details are shown in Appendix 1a.

**Table 1:** Frequencies applying different cartilage-meniscus phenotype definitions considering **the medial compartment only**, and odds for being a case knee in the FNIH cohort.

Phenotype	Composite JSL ​+ ​Pain Case (KL2 and 3)		KL 2 (n ​= ​297)	Odds for being a composite JSL ​+ ​Pain case a^(95% confidence interval)^	Odds for being a JSL-only case b^(95% confidence interval)^	KL 3 (n ​= ​188)	Odds for being a composite JSL ​+ ​Pain case a^(95% confidence interval)^	Odds for being a JSL-only case b^(95% confidence interval)^
	No	Yes						
Cartilage-meniscus Phenotype Group (D1a) - medial cartilage damage present, no more than MOAKS 2.2 c								
Has phenotype	233 (66%)	122 (34%)	191/297 (64%)	2.52 (1.40,4.54)	4.17 (2.44, 7.14)	164/188 (87%)	0.32 (0.13, 0.78)	0.48 (0.19, 1.21)
Does not have phenotype - too much damage	12 (43%)	16 (57%)	5/297 (2%)	Reference		23/188 (12%)	Reference	
Does not have phenotype - no damage	85 (83%)	17 (17%)	101/297 (34%)			1/188 (1%)		
Cartilage–meniscus phenotype Group (D2a) - medial cartilage damage present, no more than MOAKS 2.1 c								
Has phenotype	193 (67%)	93 (33%)	183/297 (62%)	1.93 (1.11,3.35)	3.30 (1.99, 5.49)	103/188 (55%)	0.56 (0.31, 1.01)	0.39 (0.21, 0.71)
Does not have phenotype - too much damage	52 (54%)	45 (46%)	13/297 (4%)	Reference		84/188 (45%)	Reference	
Does not have phenotype - no damage	85 (83%)	17 (17%)	101/297 (34%)			1/188 (1%)		
Cartilage Medial Phenotype Group (D3a) - medial cartilage damage present, no more than MOAKS 2.0 c								
Has phenotype	166 (68%)	78 (32%)	167/297 (56%)	1.92 (1.13,3.28)	2.73 (1.68, 4.42)	77/188 (41%)	0.49 (0.26, 0.91)	1.15 (0.65, 2.06)
Does not have phenotype - too much damage	79 (57%)	60 (43%)	29/297 (10%)	Reference		110/188 (59%)	Reference	
Does not have phenotype - no damage	85 (83%)	17 (17%)	101/297 (34%)			1/188 (1%)		

a Composite JSL ​+ ​Pain case definition: radiographic and pain progression.

b JSL-joint space loss, i.e. radiographic progression only.

c Any lateral cartilage and any meniscal status allowed.

Considering the medial and lateral compartments combined frequencies of those knees fulfilling a phenotype definition and those that did not due to either “too much” or “no” damage are presented in Appendix 1b The odds for being a composite case were 2.13 (95% CI 1.27, 3.59) for D1, 1.91 (95% CI 1.12, 3.26) for D2 and 1.81 (95%CI 1.03, 3.19) for the D3 definition for KL2 knees (Appendix 2). For KL3 knees odds were 1.12 (0.62, 2.02) for D1, 1.00 (0.51, 1.97) for D2 and 0.90 (0.41, 1.97) for D3. For the JSL-only outcome, increased odds for KL2 knees for definitions D1 and D2 but not D3 were observed and for KL3 knees no statistically significant results were seen **(**Appendix 2**)**.

Regarding frequencies of the different meniscus-cartilage phenotypes, for KL2 and 3 knees combined, numbers increased from 5% using the original ROAMES definition [4] and 21% using the modified published definition [5] to 73%, 59% and 50% for medial cartilage damage only (Table 2a). Including any ipsicompartmental meniscal damage to the definitions of a cartilage-meniscus phenotype defined by changes in the medial compartment only, these numbers changed to 45%, 33% and 27% (Table 2b). Associations between phenotype and progression (composite JSL ​+ ​pain case, JSL-only case) were generally similar between definitions with (Appendix 3) and without (Table 1) meniscal involvement.

**Table 2a:** Frequencies of knees using different cartilage/meniscus phenotype definitions based on compartmental cartilage thresholds.

Presence of phenotype	KLG 2	KLG 3	KL 2 ​+ ​3 combined
Cartilage/meniscus Phenotype (Original ROAMES) a			
No	291 (98%)	172 (91%)	463 (95%)
Yes	6 (2%)	16 (9%)	22 (5%)
Cartilage/meniscus Phenotype (secondary published definition modified ROAMES) b			
No	282 (95%)	99 (53%)	381 (79%)
Yes	15 (5%)	89 (47%)	104 (21%)
Cartilage Medial Phenotype (D1a) c- med cartilage damage present, no more than 2.2			
No	106 (36%)	24 (13%)	130 (27%)
Yes	191 (64%)	164 (87%)	355 (73%)
Cartilage Medial Phenotype (D2a) c- med cartilage damage present, no more than 2.1			
No	114 (38%)	85 (45%)	199 (41%)
Yes	183 (62%)	103 (55%)	286 (59%)
Cartilage Medial Phenotype (D3a) c- med cartilage damage present, no more than 2.0			
No	130 (44%)	111 (59%)	241 (50%)
Yes	167 (56%)	77 (41%)	244 (50%)
Cartilage Medial and Lateral Phenotype (D1b) d - med and lat cartilage damage present, no more than 2.2			
No	192 (65%)	105 (56%)	297 (61%)
Yes	105 (35%)	83 (44%)	188 (39%)
Cartilage Medial and Lateral Phenotype (D2b) d - med and lat cartilage damage present, no more than 2.1			
No	209 (70%)	141 (75%)	350 (72%)
Yes	88 (30%)	47 (25%)	135 (28%)
Cartilage Medial and Lateral Phenotype (D3b) d - med and lat cartilage damage present, no more than 2.0			
No	225 (76%)	155 (82%)	380 (78%)
Yes	72 (24%)	33 (18%)	105 (22%)

a Presence of a meniscus score of at least ROAMES grade 3 (i.e., any type of meniscal substance loss/maceration) in the medial and/or lateral compartment and at least ROAMES grade 1 (any type of tear) in the other compartment, respectively, and presence of cartilage damage grades 2.1, 2.2, 3.2 or 3.3 according to MOAKS [3].

b Any type of meniscal substance loss/maceration regardless of other compartment AND presence of ipsi-compartmental cartilage damage grades ≥2.1 [4].

c For phenotypes cartilage (D1a), (D2a), and (D3a): any medial/lateral meniscal score allowed, any cartilage score allowed in the lateral compartment.

d For phenotypes cartilage (D1b), (D2b), and (D3b): any medial/lateral meniscal score allowed.

**Table 2b:** Frequencies of knees using different cartilage-meniscus phenotype definitions based on medial cartilage thresholds and concomitant ipsicompartmental presence of any or severe medial meniscal damage.

Presence of phenotype	KLG 2	KLG 3	KL 2 ​+ ​3 combined
Cartilage Medial and Meniscus Medial Phenotype (D1c) - med cartilage damage present, no more than 2.2, medial meniscus ROAMES ≥1 a			
No	213 (72%)	53 (28%)	266 (55%)
Yes	84 (28%)	135 (72%)	219 (45%)
Cartilage Medial and Meniscus Medial Phenotype (D2c) - med cartilage damage present, no more than 2.1, medial meniscus ROAMES ≥1 a			
No	216 (73%)	107 (57%)	323 (67%)
Yes	81 (27%)	81 (43%)	162 (33%)
Cartilage Medial and Meniscus Medial Phenotype (D3c) - med cartilage damage present, no more than 2.0, medial meniscus ROAMES ≥1 a			
No	222 (75%)	131 (70%)	353 (73%)
Yes	75 (25%)	57 (30%)	132 (27%)
Cartilage Medial and Severe Meniscus Medial Phenotype (D1d) - med cartilage damage present, no more than 2.2, medial meniscus ROAMES ​= ​3 b			
No	270 (91%)	82 (44%)	352 (73%)
Yes	27 (9%)	106 (56%)	133 (27%)
Cartilage Medial and Severe Meniscus Medial Phenotype (D2d) - med cartilage damage present, no more than 2.1, medial meniscus ROAMES ​= ​3 b			
No	271 (91%)	125 (66%)	396 (82%)
Yes	26 (9%)	63 (34%)	89 (18%)
Cartilage Medial and Severe Meniscus Medial Phenotype (D3d) - med cartilage damage present, no more than 2.0, medial meniscus ROAMES ​= ​3 b			
No	274 (92%)	145 (77%)	419 (86%)
Yes	23 (8%)	43 (23%)	66 (14%)

a ROAMES ≥1: any meniscal tear and/or any meniscal maceration.

b ROAMES ​= ​3: any meniscal substance loss i.e. meniscal partial or complete maceration.

## 4 Discussion

Adapting the thresholds of cartilage damage and including or excluding meniscal damage for different definitions of a cartilage-meniscus phenotype leads to increased frequencies of knees fulfilling those definitions of up to 64% for KL2 knees and 87% for KL3 knees depending on the definition and considering the medial compartment only. Increased odds for being a composite or JSL case at 48 months were seen for KL2 knees for all three definitions compared to those knees not fulfilling the definition. For KL3 using the medial compartment-only definitions, a seemingly protective effect was observed particularly for using the composite case outcome. One third of knees with KL2 (vs. 1% in KL3 knees) did not show any medial cartilage damage.

Reasons for failure of DMOAD trials are complex but include radiography-based eligibility screening to define structural disease severity [17]. X-ray is only able to depict osseous changes at relatively late stages and radiography has shortcomings regarding reproducible image acquisition [18]. MRI in contrast, is an instrument that has matured over recent years and is likely able to replace X-ray-based screening efforts [4,19]. Using phenotypic definitions based on structure may help in further stratifying suitable patient populations [20]. While particularly three different structural phenotypes have been introduced, limitations include overlap of phenotypes and that more than one may be present in an individual [5]. In addition, such phenotypic definitions as in our current study are based on *a priori* hypotheses and whether other relevant structural phenotypic definitions may exist has not been agnostically evaluated. In a systematic meta-analysis Deveza and colleagues described significant heterogeneity across studies in the selection of participants and characteristics and methods used to investigate knee OA phenotypes [21]. Furthermore, we acknowledge that a wide range of views exist on how best to operationalize the concept of OA phenotypes. The term phenotype as used in our study does not fulfill the characteristics as suggested by others and may potentially be adapted to structural “subtype” [2].

Limitations of our study include the retrospective aspect of our work analyzing a sample that has been defined by certain outcomes. We acknowledge that the matching of cases and controls has been broken up by excluding knees with posterior meniscal root tears and KL1 knees. However, we could also show that KL1 knees and those with posterior root tears were evenly distributed across the different outcome groups and should not have influenced our analysis. We did not screen the remaining knees for other diagnoses of exclusion such as subchondral insufficiency fractures or others, as we used available MOAKS readings that did not include these diagnoses.

While in a previous analysis we showed that only the subchondral bone phenotype was associated with an increased risk of progression [5], we could show in our current study that using different definitions of a cartilage-meniscus phenotype will lead to an increased risk for progression for KL2 knees compared to those knees that do not fulfill that phenotype. While for KL2 knees, the number of those excluded because they did not show any cartilage damage was high, for KL3 knees this was the case for those with too much damage i.e. severe wide-spread full-thickness cartilage loss. While an accelerated progression of cartilage loss has been shown for more severe structural OA using quantitative MRI approaches, likely knees with “too much” full-thickness damage are not ideally suited to be included particularly using anti-catabolic DMOAD approaches.

In summary, we could show that phenotypic stratification of the cartilage-meniscus phenotype in different subtypes is feasible and may help in defining trial cohorts at screening. Increased odds for progression are seen for KL2 knees and all definitions, while a seemingly protective effect is seen for KL3 knees. The latter can be explained by the fact that KL3 knees stratified by the suggested definitions have comparably mild cartilage damage at screening. One-third of knees with KL2 do not have medial cartilage damage, which is an important finding and needs to be considered when selecting patients for clinical trials based on X-ray assessment only.

## Authors contributions

Analysis and interpretation of the data: FWR, JEC, SD, DJH, AG, Drafting of the article: FWR, JEC, SD, DJH, AG Provision of study materials or patients: FWR, JEC, SD, DJH, AG Statistical expertise: JEC Obtaining funding: FWR, DJH, AG Collection and assembly of data: FWR, JEC, SD, DJH, AG Responsibility for the integrity of the work as a whole, from inception to finished article, is taken by F. Roemer, MD (first author; frank.roemer@uk-erlangen.de; froemer@bu.edu).

## Funding and role of the funding source

Scientific and financial support for the Foundation for the National Institutes of Health (FNIH) Osteoarthritis (OA) Biomarkers Consortium and for this study has been made possible through grants as well as direct and indirect in-kind contributions from AbbVie, 10.13039/100002429Amgen Inc., the 10.13039/100000980Arthritis Foundation, Bioiberica SA, 10.13039/100008426DePuy Mitek, Inc., Flexion Therapeutics, Inc., 10.13039/100004330GlaxoSmithKline, Merck Serono, Rottapharm | Madaus, Sanofi, Stryker, and the Pivotal Osteoarthritis Initiative Magnetic Resonance Imaging Analyses (POMA) Study (NIH/National Heart, Lung, and Blood Institute grant HHSN- 2682010000). The Osteoarthritis Initiative (OAI) is a public–private partnership between the NIH (contracts N01-AR-2-2258, N01-AR-2- 2259, N01-AR-2-2260, N01-AR-2-2261, and N01-AR-2-2262) and private funding partners (Merck Research Laboratories, Novartis Pharmaceuticals, GlaxoSmithKline, and 10.13039/100004319Pfizer, Inc.) and is conducted by the OAI Study Investigators. Private sector funding for the Biomarkers Consortium and the OAI is managed by the FNIH.

## Declaration of competing interest

AG has received consultancies, speaking fees, and/or honoraria from Pfizer, Novartis, AstraZeneca, Merck Serono, and TissueGene and is President and shareholder of Boston Imaging Core Lab (BICL), LLC a company providing image assessment services.

FWR is Chief Medical Officer and shareholder of BICL, LLC. and has received consultancies, speaking fees, and/or honoraria from Calibr –California Institute of Biomedical Research and Grünenthal, GmbH.

DJH is funded by an 10.13039/501100000925NHMRC Practitioner Fellowship and received a grant from the FNIH OA Biomarkers Consortium. He reports consulting fees from Merck Serono, TLCBio, Pfizer and Lilly.
