# Supplementary material for: Patterns of progression differ between Kellgren-Lawrence 2 and 3 knees fulfilling different definitions of a cartilage-meniscus phenotype in the Foundation for National Institutes of Health Osteoarthritis Biomarkers study (FNIH)
Source: Osteoarthr Cartil Open. 2022 Jun 9;4(3):100284. doi: 10.1016/j.ocarto.2022.100284 (PMC9718096; doi:10.1016/j.ocarto.2022.100284)
Supplement: Multimedia component 1 [file mmc1.docx]

**Appendix 1 (a).** Frequencies applying different cartilage-meniscus phenotype definitions considering **the medial compartment only**, and frequency for being a case knee in the FNIH cohort by KLG

| **Phenotype** | KLG2 (n=297) | | | |  | KLG3 (n=188) | | | |
| --- | --- | --- | --- | --- | --- | --- | --- | --- | --- |
|  | Composite  JSL + Pain Case^1^ | | JSL-only case^2^ | |  | Composite  JSL + Pain Case^1^ | | JSL-only case^2^ | |
|  | No | Yes | No | Yes |  | No | Yes | No | Yes |
| Cartilage-meniscus Phenotype Group (D1a) - medial cartilage damage present, no more than MOAKS 2.2^3^ | | | | | | | | | |
| Has phenotype | 126 (66%) | 65 (34%) | 86 (45%) | 105 (55%) |  | 107 (65%) | 57 (35%) | 76 (46%) | 88 (54%) |
| Does not have phenotype - too much damage | 4 (80%) | 1 (20%) | 4 (80%) | 1 (20%) |  | 8 (35%) | 15 (65%) | 6 (26%) | 17 (74%) |
| Does not have phenotype - no damage^3^ | 84 (83%) | 17 (17%) | 78 (77%) | 23 (23%) |  | 1 (100%) | 0 (0%) | 1 (100%) | 0 (0%) |
| Cartilage–meniscus phenotype Group (D2a) - medial cartilage damage present, no more than MOAKS 2.1^3^ | | | | | | | | | |
| Has phenotype | 123 (67%) | 60 (33%) | 84 (46%) | 99 (54%) |  | 70 (68%) | 33 (32%) | 56 (54%) | 47 (46%) |
| Does not have phenotype - too much damage | 7 (54%) | 6 (46%) | 6 (46%) | 7 (54%) |  | 45 (54%) | 39 (46%) | 26 (31%) | 58 (69%) |
| Does not have phenotype - no damage^3^ | 84 (83%) | 17 (17%) | 78 (77%) | 23 (23%) |  | 1 (100%) | 0 (0%) | 1 (100%) | 0 (0%) |
| Cartilage Medial Phenotype Group (D3a) - medial cartilage damage present, no more than MOAKS 2.0^3^ | | | | | | | | | |
| Has phenotype | 111 (66%) | 56 (34%) | 77 (46%) | 90 (54%) |  | 55 (71%) | 22 (29%) | 47 (61%) | 30 (39%) |
| Does not have phenotype - too much damage | 19 (66%) | 10 (34%) | 13 (45%) | 16 (55%) |  | 60 (55%) | 50 (45%) | 35 (32%) | 75 (68%) |
| Does not have phenotype - no damage^3^ | 84 (83%) | 17 (17%) | 78 (77%) | 23 (23%) |  | 1 (100%) | 0 (0%) | 1 (100%) | 0 (0%) |

^1^ Composite JSL + Pain case definition: radiographic and pain progression

^2^ JSL- joint space loss, i.e. radiographic progression only

^3^ “no damage” meaning either combined absence of damage medial AND lateral, or absence of damage in one compartment AND damage in the other compartment not more than MOAKS 2.0 (Definition 1a), not more than MOAKS 2.1 (Definition 2a) or not more than MOAKS 2.0 (Definition 3a)

^4^ any lateral cartilage and any meniscal status allowed

**Appendix 1 (b).** Frequencies applying different cartilage-meniscus phenotype definitions considering concomitant cartilage damage in **the medial and lateral compartment**, and frequency for being a case knee in the FNIH cohort by KLG

| **Phenotype** | KLG2 | | | |  | KLG3 | | | |
| --- | --- | --- | --- | --- | --- | --- | --- | --- | --- |
|  | Composite  JSL + Pain Case^1^ | | JSL-only case^2^ | |  | Composite  JSL + Pain Case^1^ | | JSL-only case^2^ | |
|  | No | Yes | No | Yes |  | No | Yes | No | Yes |
| Cartilage-meniscus Phenotype Group (D1b) - medial and lateral cartilage damage present, no more than MOAKS 2.2 ^4^ | | | | | | | | | |
| Has phenotype | 65 (62%) | 40 (38%) | 45 (43%) | 60 (57%) |  | 50 (60%) | 33 (40%) | 35 (42%) | 48 (58%) |
| Does not have phenotype - too much damage | 5 (71%) | 2 (29%) | 4 (57%) | 3 (43%) |  | 8 (35%) | 15 (65%) | 6 (26%) | 17 (74%) |
| Does not have phenotype - no damage^3^ | 144 (78%) | 41 (22%) | 119 (64%) | 66 (36%) |  | 58 (71%) | 24 (29%) | 42 (51%) | 40 (49%) |
| Cartilage–meniscus phenotype Group (D2b) - medial and lateral cartilage damage present, no more than MOAKS 2.1 ^4^ | | | | | | | | | |
| Has phenotype | 55 (63%) | 33 (38%) | 39 (44%) | 49 (56%) |  | 29 (62%) | 18 (38%) | 22 (47%) | 25 (53%) |
| Does not have phenotype - too much damage | 20 (63%) | 12 (38%) | 15 (47%) | 17 (53%) |  | 50 (56%) | 39 (44%) | 31 (35%) | 58 (65%) |
| Does not have phenotype - no damage^3^ | 139 (79%) | 38 (21%) | 114 (64%) | 63 (36%) |  | 37 (71%) | 15 (29%) | 30 (58%) | 22 (42%) |
| Cartilage Medial Phenotype Group (D3b) - medial and lateral cartilage damage present, no more than MOAKS 2.0 ^4^ | | | | | | | | | |
| Has phenotype | 45 (63%) | 27 (38%) | 34 (47%) | 38 (53%) |  | 21 (64%) | 12 (36%) | 18 (55%) | 15 (45%) |
| Does not have phenotype - too much damage | 41 (66%) | 21 (34%) | 28 (45%) | 34 (55%) |  | 65 (57%) | 50 (43%) | 39 (34%) | 76 (66%) |
| Does not have phenotype - no damage^3^ | 128 (79%) | 35 (21%) | 106 (65%) | 57 (35%) |  | 30 (75%) | 10 (25%) | 26 (65%) | 14 (35%) |

^1^ Composite JSL + Pain case definition: radiographic and pain progression

^2^ JSL- joint space loss, i.e. radiographic progression only

^3^ “no damage” meaning either combined absence of damage medial AND lateral, or absence of damage in one compartment AND damage in the other compartment not more than MOAKS 2.0 (Definition 1b), not more than MOAKS 2.1 (Definition 2b) or not more than MOAKS 2.0 (Definition 3b)

^4^ any medial and lateral meniscal status allowed

**Appendix 2.** Frequencies applying different cartilage-meniscus phenotype definitions considering concomitant cartilage damage in **the medial and lateral compartment** and odds for being a case knee in the FNIH cohort

| Phenotype | Composite JSL + Pain Case (KL2 and 3) | | KL 2 | Odds for being a composite JSL + Pain case ^1^  ^(95% confidence interval)^ | Odds for being a JSL-only case ^2^  ^(95% confidence interval)^ | KL 3 | Odds for being a composite JSL + Pain case ^1^  ^(95% confidence interval)^ | Odds for being a JSL-only case ^2^  ^(95% confidence interval)^ |
| --- | --- | --- | --- | --- | --- | --- | --- | --- |
|  | No | Yes |  |  |  |  |  |  |
| Cartilage-meniscus Phenotype Group (D1b) - medial and lateral cartilage damage present, no more than MOAKS 2.2 ^4^ | | | | | | | | |
| Has phenotype | 115 (61%) | 73 (39%) | 105 (35%) | 2.13 (1.27, 3.59) | 2.38 (1.46, 3.86) | 83 (44%) | 1.12 (0.62, 2.02) | 1.15 (0.65, 2.06) |
| Does not have phenotype - too much damage | 13 (43%) | 17 (57%) | 7 (2%) | Reference | | 23 (12%) | Reference | |
| Does not have phenotype - no damage^3^ | 202 (76%) | 65 (24%) | 185 (62%) |  |  | 82 (44%) |  |  |
| Cartilage–meniscus phenotype Group (D2b) - medial and lateral cartilage damage present, no more than MOAKS 2.1 ^4^ | | | | | | | | |
| Has phenotype | 84 (62%) | 51 (38%) | 88 (30%) | 1.91 (1.12, 3.26) | 2.03 (1.22, 3.36) | 47 (25%) | 1.00 (0.51, 1.97) | 0.87 (0.45, 1.68) |
| Does not have phenotype - too much damage | 70 (58%) | 51 (42%) | 32 (11%) | Reference | | 89 (47%) | Reference | |
| Does not have phenotype - no damage^3^ | 176 (77%) | 53 (23%) | 177 (60%) |  |  | 52 (28%) |  |  |
| Cartilage Medial Phenotype Group (D3b) - medial and lateral cartilage damage present, no more than MOAKS 2.0 ^4^ | | | | | | | | |
| Has phenotype | 66 (63%) | 39 (37%) | 72 (24%) | 1.81 (1.03, 3.19) | 1.65 (0.97, 2.81) | 33 (18%) | 0.90 (0.41, 1.97) | 0.60 (0.28, 1.28) |
| Does not have phenotype - too much damage | 106 (60%) | 71 (40%) | 62 (21%) | Reference | | 115 (61%) | Reference | |
| Does not have phenotype - no damage^3^ | 158 (78%) | 45 (22%) | 163 (55%) |  |  | 40 (21%) |  |  |

^1^ Composite JSL + Pain case definition: radiographic and pain progression

^2^ JSL- joint space loss, i.e. radiographic progression only= Original Table 2; renamed to Appendix 2= Original Table 2; renamed to Appendix 2

^3^ “no damage” meaning either combined absence of damage medial AND lateral, or absence of damage in one compartment AND damage in the other compartment not more than MOAKS 2.0 (Definition 1b), not more than MOAKS 2.1 (Definition 2b) or not more than MOAKS 2.0 (Definition 3b)

^4^ any medial and lateral meniscal status allowed

**Appendix 3.** Frequencies applying different cartilage-meniscus phenotype definitions based on **medial cartilage thresholds** and concomitant **ipsicompartmental presence of any or severe medial meniscal damage** and odds for being a case knee in the FNIH cohort

| Phenotype | Composite JSL + Pain Case ^1^ (KL2 and 3) | | KL 2 | Odds for being a composite JSL + Pain case ^1^  ^(95% confidence interval)^ | Odds for being a JSL-only case ^2^  ^(95% confidence interval)^ | | KL 3 | Odds for being a composite JSL + Pain case ^1^  ^(95% confidence interval)^ | | Odds for being a JSL-only case ^2^  ^(95% confidence interval)^ |
| --- | --- | --- | --- | --- | --- | --- | --- | --- | --- | --- |
|  | No | Yes |  |  |  |  |  |  |  |  |
| Cartilage Medial and Meniscus Medial Phenotype (D1c) - med cartilage damage present, no more than 2.2, medial meniscus ROAMES ≥1 ^3^ | | | | | | | | | | |
| No | 187 (70%) | 79 (30%) | 213 (72%) | 1.44 (0.83, 2.48) | 3.84 (2.25, 6.55) | | 53 (28%) | 0.67 (0.35, 1.27) | | 1.07 (0.56, 2.02) |
| Yes | 143 (65%) | 76 (35%) | 84 (28%) | Reference | | | 135 (72%) | Reference | | |
| Cartilage Medial and Meniscus Medial Phenotype (D2c) - med cartilage damage present, no more than 2.1, medial meniscus ROAMES ≥1 ^3^ | | | | | | | | | | |
| No | 220 (68%) | 103 (32%) | 216 (73%) | 1.32 (0.76, 2.30) | 3.49 (2.04, 5.96) | | 107 (57%) | 0.63 (0.34, 1.15) | | 0.58 (0.32, 1.04) |
| Yes | 110 (68%) | 52 (32%) | 81 (27%) | Reference | | | 81 (43%) | Reference | | |
| Cartilage Medial and Meniscus Medial Phenotype (D3c) - med cartilage damage present, no more than 2.0, medial meniscus ROAMES ≥1 ^3^ | | | | | | | | | | |
| No | 237 (67%) | 116 (33%) | 222 (75%) | 1.30 (0.74, 2.30) | 3.09 (1.79, 5.34) | | 131 (70%) | 0.46 (0.23, 0.92) | | 0.36 (0.19, 0.69) |
| Yes | 93 (70%) | 39 (30%) | 75 (25%) | Reference | | | 57 (30%) | Reference | | |
| Cartilage Medial and Severe Meniscus Medial Phenotype (D1d) - med cartilage damage present, no more than 2.2, medial meniscus ROAMES =3 ^4^ | | | | | | | | | | |
| No | 237 (67%) | 115 (33%) | 270 (91%) | 0.72 (0.28, 1.84) | | 2.40 (1.06, 5.43) | 82 (44%) | 0.55 (0.30, 0.99) | 0.90 (0.50, 1.61) | |
| Yes | 93 (70%) | 40 (30%) | 27 (9%) | Reference | | | 106 (56%) | Reference | | |
| Cartilage Medial and Severe Meniscus Medial Phenotype (D2d) - med cartilage damage present, no more than 2.1, medial meniscus ROAMES =3 ^4^ | | | | | | | | | | |
| No | 263 (66%) | 133 (34%) | 271 (91%) | 0.59 (0.21, 1.62) | | 2.24 (0.98, 5.11) | 125 (66%) | 0.47 (0.24, 0.91) | 0.55 (0.30, 1.01) | |
| Yes | 67 (75%) | 22 (25%) | 26 (9%) | Reference | | | 63 (34%) | Reference | | |
| Cartilage Medial and Severe Meniscus Medial Phenotype (D3d) - med cartilage damage present, no more than 2.0, medial meniscus ROAMES =3 ^4^ | | | | | | | | | | |
| No | 277 (66%) | 142 (34%) | 274 (92%) | 0.70 (0.25, 1.95) | | 2.15 (0.90, 5.14) | 145 (77%) | 0.29 (0.13, 0.67) | 0.29 (0.14, 0.59) | |
| Yes | 53 (80%) | 13 (20%) | 23 (8%) | Reference | | | 43 (23%) | Reference | | |

^1^ Composite JSL + Pain case definition: radiographic and pain progression

^2^ JSL- joint space loss, i.e. radiographic progression only

^3^ ROAMES ≥1: any meniscal tear and/or any meniscal maceration

^4^ ROAMES = 3: any meniscal substance loss i.e. meniscal partial or complete maceration
